# Intentional co-ingestion of levothyroxine and insulin in a teenager

**DOI:** 10.1007/s40200-026-01877-9

**Published:** 2026-02-21

**Authors:** Sarah El Yaman, Kathryn Swaby, Jeffrey N. Bernstein, Janine E. Sanchez

**Affiliations:** 1 Department of Pediatric Endocrinology, University of Miami/Jackson Health System, Miami, FL USA; 2 Department of Pediatric Critical Care Medicine, University of Miami/Jackson Health System, FL Miami, USA; 3 Department of Emergency Medicine, Medical Toxicology, University of Miami/Jackson Health System, Miami, FL USA

**Keywords:** Levothyroxine, Insulin, Thyrotoxicosis, Overdose, Adolescent

## Abstract

**Purpose:**

To report a case of polydrug ingestion involving levothyroxine and insulin in an adolescent, demonstrating potential protective effects of insulin co-ingestion against symptomatic thyrotoxicosis.

**Methods:**

A 14-year-old female with type 1 diabetes and Hashimoto thyroiditis presented following intentional ingestion of 6.75 milligrams levothyroxine, 60 units insulin aspart, and 80 units insulin degludec. Laboratory studies revealed severe biochemical thyrotoxicosis with free thyroxine greater than 7.77 nanograms per deciliter and total triiodothyronine 220.5 nanograms per deciliter. She required glucose infusion at 2.3 to 3.7 milligrams per kilogram per minute to maintain normoglycemia, with hypoglycemic episodes lasting up to 17 h post-ingestion. Throughout her 12-day admission, she maintained normal vital signs with no clinical evidence of sympathetic overactivity despite prolonged biochemical thyrotoxicosis.

**Conclusions:**

Co-ingestion of insulin and levothyroxine appears to minimize symptomatic thyrotoxicosis risk, potentially through inhibition of thyroxine to triiodothyronine conversion and glucose homeostasis maintenance. This case highlights complex interactions in polydrug ingestions and suggests insulin may provide protection against thyroid storm in mixed overdoses.

## Introduction

Intentional drug overdoses with multiple agents are common occurrences that often lead to greater morbidity and mortality due to drug interactions. The agents used in pediatric intentional drug ingestions are typically those readily available in the home, with outcomes that are age, weight, and drug dependent. While accidental overdoses of either levothyroxine or insulin are frequent with varied impacts, intentional insulin overdose in patients with diabetes is infrequent and potentially life-threatening [1–3]. Even rarer is the combination of high doses of both short and long-acting insulin with potentially toxic doses of levothyroxine in a suicide attempt.

## Case description

A 14-year-old female with type 1 diabetes (Islet Cell Antigen (ICA)-512 and Glutamic acid decarboxylase (GAD)-65 antibody positive), Hashimoto thyroiditis, and non-epileptic seizures presented following a suicide attempt involving polydrug ingestion. She initially reported consuming 90 tablets of 75 mcg levothyroxine (total dose 6.75 mg) and arrived at the emergency department 3 h after ingestion.

### Clinical presentation

She was well-appearing with Glasgow Coma Scale 15, temperature 37.2 °C, heart rate 78 bpm, respiratory rate 18 bpm, and blood pressure 138/95 mmHg. Point-of-care glucose was 50 mg/dl (normal 70–100 mg/dl). Her weight was 53.6 kg (BMI 18.55). She received 3 ml/kg bolus of 10% dextrose, with initial glucose improvement to 73 mg/dl, but subsequently developed recurrent hypoglycemia (nadir 38 mg/dl) requiring a second dextrose bolus and continuous infusion. Upon further questioning, she admitted to also administering the remaining contents of her insulin aspart pen (maximum 60 units) and insulin degludec pen (maximum 80 units).

### Laboratory findings

Initial laboratory studies revealed: FT4 > 7.77 ng/dl (normal 0.7–1.9 ng/dl), TT3 220.5 ng/dl (normal 102–200 ng/dl), thyroid stimulating hormone 5.25 µIU/ml (normal 0.5–4.70 µIU/ml), insulin level 0.4 µU/ml (normal 5–25 µU/ml), and C-peptide 0.03 ng/ml (normal 0.8-4.0 ng/ml). Complete blood count, comprehensive metabolic panel, and urine toxicology were normal. Electrocardiogram showed normal sinus rhythm.

### Clinical course

The patient was admitted to the Pediatric Intensive Care Unit, requiring glucose infusion rate of 2.3–3.7 mg/kg/min to maintain normoglycemia with recurrent hypoglycemic episodes lasting up to 17 h post-ingestion ^4–6^. She resumed prior insulin coverage on day 2 with carbohydrate ratio of 1:10 g and correction factor of 1 unit per 50 mg/dl above 200 mg/dl. Basal insulin glargine 26 units daily was resumed on day 5.

### Thyroid function monitoring

Peak thyroid hormone levels occurred on day 4 with TT3 395.2 ng/dl and FT4 5.25 ng/dl. Levels gradually normalized over 30 days, with FT4 returning to 1.04 ng/dl and TSH recovering to 3.58 µIU/ml by discharge.

### Clinical outcome

Throughout her 12-day admission, the patient maintained normal heart rate (56–88 bpm) and blood pressure with no clinical evidence of sympathetic overactivity despite prolonged biochemical thyrotoxicosis. She experienced only heat intolerance, with no rhythm disturbances, tremulousness, diaphoresis, seizures, fever, diarrhea, abdominal pain, or mental status changes. No beta-adrenergic blockers or antithyroid medications were administered.

## Conclusion

This case represents a rare combination of intentional overdose with both insulin and levothyroxine, resulting in asymptomatic thyrotoxicosis despite significant biochemical abnormalities. The patient’s clinical presentation contrasts markedly with typical expectations for either drug overdose alone.

Intentional insulin overdose typically causes prolonged hypoglycemia lasting up to 70 h, with manifestations ranging from asymptomatic to comatose states requiring mechanical ventilation [1–6]. Prognosis depends more on time to presentation than absolute insulin dose. Complications include electrolyte disturbances, pulmonary edema, hepatic dysfunction, and permanent cognitive impairment [4].

Levothyroxine overdose presentations vary from completely asymptomatic despite biochemical thyrotoxicosis to comatose with tachyarrhythmias and heart failure [7–11]. Thyroid storm is characterized by rapidly rising temperature, tachycardia progressing to atrial arrhythmias, widened pulse pressure, extreme agitation, and diarrhea [9]. Most reported acute L-thyroxine ingestions occur in children, with serious sequelae being rare [9–11]. The rarity of symptomatic pediatric L-thyroxine ingestions has been attributed to greater shunting of T3 to biologically inactive reverse T3, maintaining a euthyroid state [11].

Our literature search revealed only one previous case report of insulin and thyroxine co-ingestion in a 46-year-old diabetic man who developed severe hypoglycemia with persistent tachycardia requiring beta-adrenergic blockers [12]. Peak hormone levels were similar to our patient, but the clinical presentation differed significantly.

The asymptomatic presentation in our patient may be explained by several mechanisms. Elevated insulin levels can interfere with T4 to T3 conversion through suppression or competitive inhibition of deiodinase enzymes, favoring production of reverse T3 (less biochemically active) [13]– [14]. This provides a protective mechanism against thyrotoxicosis. Additionally, thyrotoxicosis increases glucose absorption, decreases muscle glycogen storage, and increases hepatic gluconeogenesis, potentially helping maintain blood glucose levels [13] High-dose insulin may provide cardioprotective effects by improving cardiac myocyte function and enhancing vasodilation, which helps reverse cardiogenic shock in cases of beta-blocker and calcium channel-blocker poisoning [15].

The co-ingestion appears to create a unique pharmacological interaction where insulin mitigates thyrotoxicosis symptoms while levothyroxine helps maintain glucose homeostasis, resulting in a relatively benign clinical course despite significant biochemical abnormalities.

## Data Availability

No datasets were generated or analysed during the current study.
